# Impact of data on generalization of AI for surgical intelligence applications

**DOI:** 10.1038/s41598-020-79173-6

**Published:** 2020-12-17

**Authors:** Omri Bar, Daniel Neimark, Maya Zohar, Gregory D. Hager, Ross Girshick, Gerald M. Fried, Tamir Wolf, Dotan Asselmann

**Affiliations:** 1 theator Inc., San Mateo, CA USA; 2 Department of Computer Science, Johns Hopkins University, Baltimore, USA; 3 Department of Surgery, McGill University, Montreal, QC Canada

**Keywords:** Endoscopy, Endoscopy, Translational research, Biomedical engineering, Electrical and electronic engineering, Computer science, Scientific data

## Abstract

AI is becoming ubiquitous, revolutionizing many aspects of our lives. In surgery, it is still a promise. AI has the potential to improve surgeon performance and impact patient care, from post-operative debrief to real-time decision support. But, *how much data is needed by an AI-based system to learn surgical context with high fidelity?* To answer this question, we leveraged a large-scale, diverse, cholecystectomy video dataset. We assessed surgical workflow recognition and report a deep learning system, that not only detects surgical phases, but does so with high accuracy and is able to generalize to new settings and unseen medical centers. Our findings provide a solid foundation for translating AI applications from research to practice, ushering in a new era of surgical intelligence.

## Introduction

Surgery is an indispensable element to the treatment of disease. It is estimated that 312.9 million major surgical procedures were performed worldwide in 2012, including 36 million in the United States alone^1^. To put this in context, during an average 85 year lifespan it has been estimated that the average American will undergo more than nine surgical procedures^2^. It is also well-established that the quality of surgeon performance has a critical impact on the quality and overall cost of care, with poor performance resulting in substantial increases in morbidity, reoperation, and mortality^3–5^. Yet, much of surgical training and credentialing is still performed as it has been for the past 100 years—namely, through mentored one-on-one graduated training^6,7^.

Recent advances in artificial intelligence (AI) hold potential to enhance surgical training, improve surgeon performance and ultimately surgical outcomes. This is particularly true for minimally invasive surgery (MIS). In general surgery for example, the MIS surgeon operates by viewing the surgical field via a live video stream acquired by a laparoscope, inserted into the abdomen. In these, as well as robotic procedures, most essential aspects of surgery are captured by video feed. It has also been established that certain critical parameters of surgical performance can be measured from video by human observers^3^ these metrics can be used to predict patient outcomes. Taken together, this suggests that AI-based systems that are able to provide real-time recommendations during surgery have great potential to support surgeons’ training and their decision-making process^8^ potentially reducing the risk for complications and improving outcomes^9,10^.

A key challenge for machine-learning (ML)-driven AI is the need for correctly labeled, representative data to drive the learning process. Medicine in general, and surgery in particular, present unique challenges to the capture, labeling, and sharing of data^10^. As a result, nearly every study of machine learning applied to surgical performance has been on a small (typically far less than 100) video recording procedure set^11–15^. To date, the largest publicly available dataset is Cholec80, which contains 80 videos of laparoscopic cholecystectomies performed by 13 surgeons from a single medical center^11^. Given that it is well established that even human surgeons require well more the 100 repetitions of the same surgery to reach expert performance levels^16–18^ and the fact that typical video classification models in other domains are trained on more than 100,000 videos^19,20^, it seems unlikely the results reported on a few dozen videos represent the true potential of AI for surgery.

In this study, we focus on answering two key questions fundamental to the application of AI to surgery: (1) *How many surgical video recordings are necessary to train an AI system to recognize the major phases of a surgical procedure?* and (2) *How robust is the learned model to new sources (surgeon and/or medical centers) of data?* To answer these questions, we report the results of state-of-the-art ML on a dataset that contains 1243 videos of laparoscopic cholecystectomy—an order of magnitude (10 times) larger than all previously published studies.

We evaluated our ability to train a system to learn surgical context using the benchmark task of surgical phase recognition—that is, segmenting a video of surgery into the major steps that constitute the procedure. Phase recognition is a foundational step in automating surgical analysis applications and was used previously in surgical modeling challenges^21^. The task of surgical phase recognition is similar to the task of general action recognition^19,22,23^ but is more challenging due to the need to correctly label every second of surgery with a phase of the task, e.g. Dissection, Division, Separation, Packaging, and so forth. It is essential for predicting the next surgical task, identifying critical events timing during surgery and rapid navigation within a video, which facilitate various use cases such as post-operative debrief. Despite its benefits, routine post-operative debrief is not common. Mainly because in today’s work environment, reviewing a lengthy procedure, that just took hours to complete, is a time-consuming task that no surgeon has time for. The ability to quickly summarize a video into a palatable version, in which every major element can be quickly viewed, is a step in the right direction. Overlaying this, the recognition of particular adverse events, such as bleeding and bile spillage, and linking them to the particular phase of the procedure, will provide important analytics that can be the basis for targeted debriefing and educational interventions to enhance safety.

### The challenge of generalization

The core of our algorithmic approach is the application of recent advances in deep learning to video sequences. The dramatic impact deep learning has had on AI in general, and computer vision in particular, is strongly related to the availability of large, representative datasets. In contrast to publicly available images and videos in online repositories that contain millions of samples^19,20,24^ surgery videos are limited due to privacy and regulation issues. Additionally, the process of annotating surgical videos requires skilled personnel, making the curation process complex, slow and as a result—expensive.

The success of deep learning on image-based tasks has led to growing interest in research applying similar ideas to video-based problems. Even though the two share many key elements, the main challenge when handling videos is the addition of a temporal dimension, thus increasing the dimensionality and size of the data to be processed. A growing collection of large-scale video datasets, such as the work of Kay et al.^19^ and the work of Abu-El-Haija et al.^20^, have nonetheless enabled the development of state-of-the-art methods in video classification and action recognition tasks, where the goal is to predict a single label for a given video^22,23^.

Compared to general action recognition in video, the task of surgical phase recognition benefits from the fact that different procedures of the same surgery are similarly structured. The procedural flow, the tools used, and the surrounding anatomic context are similar no matter where the surgery is performed and who is performing it. However, in contrast, in surgical phase recognition we need to detect relatively small differences across time—a change in tool, a change in anatomic target, or difference in how a tool is used. These details are sometimes difficult to discern, even for an expert surgeon. Further, the system must do this correctly for every second of video rather than computing a single class label for an entire video. This makes it difficult to predict both the amount of data necessary to train a model for surgical phase recognition, and the robustness of the model to natural variations that will occur in practice, and thus motivates our study.

## Methods

### Definitions of laparoscopic cholecystectomy phases

Gallbladder removal, or cholecystectomy, is performed more than 750,000 times annually in the US alone, mainly for benign gallstone disease which affects 10–15% of adults^25^. The vast majority of gallbladder resections are done in relatively universal and predefined steps. These steps are the standard workflow a surgeon goes through during surgery and are known as surgical phases.

Reviewing a surgical video and classifying each of its parts into the correct phase requires a set of guiding rules. These rules define the transition between phases and the sub-tasks performed in each phase. Determining the right phase anthology is a challenging task. It is important to establish a generic set of rules which eventually reduce the potential for subjective judgment when labeling phases during surgery.

In this work, we define seven laparoscopic cholecystectomy phases: Preparation, Adhesiolysis, Dissection, Division, Separation, Packaging, and Final inspection. Although phase definition is not standardized, phases were defined based on discussions with expert surgeons and AI researchers, and validated for relevancy by an iterative process of hands-on annotations experience, taking into account clinically-relevant and algorithmically-meaningful considerations. In defining phases, we attempted to simulate a surgeon’s common workflow, focusing on the goal or action performed in each phase, and using surgical tools as cues (when possible) to support the beginning of each phase.

- *Phase 0: Preparation* obtaining pneumoperitoneum, trocar placements, optimizing exposure through patient positioning and retraction. Generally, this will be the first phase, starting once the procedure begins or as the camera entered through the trocar. However, some videos lack this phase due to a delay in starting the recording and thus start at a later phase.
- *Phase 1: Adhesiolysis* dividing adhesions. It begins when the surgical tools (like scissors or monopolar hook) start separating the adhesions. This phase is not mandatory and only performed when adhesions exist.
- *Phase 2: Dissection* dissecting hepatocystic triangle or separating gallbladder lower part from the liver bed. It begins when related surgical tools start the dissection and complete after achieving optimal visualization and skeletonization of key anatomical structures, e.g. cystic duct and cystic artery.
- *Phase 3: Division* performing cystic structures division. It starts with the introduction of a related surgical tool, such as a clip applicator, a ligature to occlude the cystic duct and artery, scissors, stapler, or even an energy device.
- *Phase 4: Separation* gallbladder dissection from the liver bed. This phase begins when a separating tool enters or, in case the tool is already present as part of the previous phase, when it starts dissecting and separating the gallbladder from the liver bed.
- *Phase 5: Packaging* gallbladder packaging. Phase starts when an endobag enters or first shown in the video stream. This phase is not mandatory as surgeons might extract the gallbladder using a strong grasping instrument.
- *Phase 6: Final inspection* a closing step when the surgeon is completing the operation by reviewing the abdominal cavity, extracting the gallbladder, removing instruments and trocars. This phase begins when any act of procedure closure is done.

Although these phases aim to follow the progression of a typical laparoscopic cholecystectomy procedure, the order of appearance might change and vary due to different reasons, e.g. phase 5 can occur after phase 6 in case the gallbladder is packed only at the end of the procedure. Moreover, the surgeon might alternate back and forth between phases, e.g. when cystic structures division is performed, the surgeon might choose to keep doing the dissection phase in order to further clear the cystic structures surrounding.

Phase definition in this work shares some common ground with the work of Twinanda et al.^11^, but we did modify a few phases after reviewing our dataset. This is done in order to provide a more generic set of guidelines. We separate phase 2 (Calot triangle dissection) of Twinanda et al.^11^ into two different phases: Adhesiolysis and Dissection. First, there is an application difference between adhesiolysis and dissection in terms of the method used by the surgeon, the patient’s condition, the excessiveness of adhesions, etc. And second, the fact that adhesions are not limited to the gallbladder and might include other organs not related to the Calot triangle. In addition, after reviewing procedures within our dataset we found that in a large number of cases gallbladder retrieval does not necessarily occur at the end of the procedure as in Cholec80^11^. This led to unnatural phase jittering between the cleaning phase and the retraction phase. In order to support this variation, we decided to combine the final visualization of the abdominal cavity and the extraction of the gallbladder as part of the final inspection phase.

We underline that these sets of guidelines were defined independently of any model training or performance analysis in order to avoid any biases.

Supplementary Table S4 illustrates phase distribution in our dataset. The phases are relatively evenly distributed, with phase 1, which is more patient-specific and thus does not always take place, having the lowest number of instances.

### Video preprocessing

All videos in this study are processed in the same manner. Initially, videos are processed using FFmpeg 3.4.6 on Ubuntu 18.04 and all video streams are encoded with libx264, using 25 frames per second (FPS). The video width is scaled to 480 and the height is determined to maintain the aspect ratio of the original input video. The audio signal is removed from all videos.

Since videos are recorded during surgery and are not edited by the staff, the original video has non-relevant segments at the beginning and end of the video file. We trim off these segments in order to avoid noise segments that are not relevant to the procedure. In order to achieve the trimmed video version, we use a background detection model. This model was trained to identify non-relevant frames that were captured outside the body. The non-relevant frames are then utilized to identify the actual start and end of the procedure in the full video and trim it down.

Preprocessing steps and the final verified video files are automatically processed and stored in a compliant, private, and secure cloud environment.

### Phase classification models

Our method, further detailed below, relies only on surgical phase labeling. As a first step, it learns from short surgery clips, taking advantage of the temporal context instead of using static 2D images, then the entire procedure is analyzed as a time series in order to learn long dependencies. In order to overcome overfitting^26^ and increase the generalization of our approach, we train on a diverse dataset and evaluate the models' robustness against several key factors.

**Figure 1:**
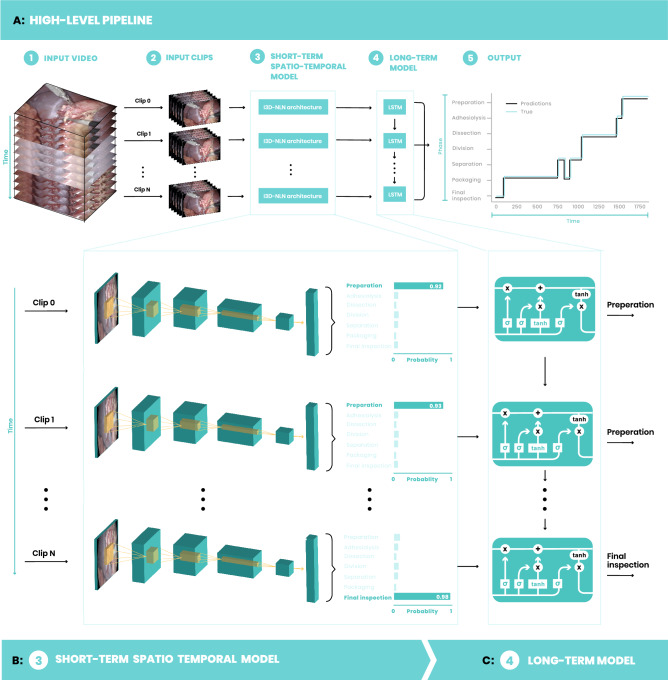
High-level flow. (**A**) The surgery input video (1) is divided into clips (2), one per second. Each clip is then fed to the short-term model which is based on an inflated 3D network with non-local blocks architecture (3). The predictions of (3) are sequentially fed to an LSTM network as part of the long-term model (4). The outputs of (4) are phase prediction for each second in the input video. Performance evaluation is done by comparing these predictions to the ground truth labels (5). The ground truth line is slightly indented in the y-axis to allow a better view of the results. (**B**) The short-term model process each clip independently to obtain a SoftMax probability vector, which represents a probability value for each phase as captured in the input clip. The probabilities are illustrated by the histograms on the right-hand side. (**C**) Probability values produced by the short-term model are applied to create a sequence of phases that are fed to a single layer LSTM network. The outputs of the long-term model are the final phase predictions.

The proposed surgical workflow phase detection system is a two-step framework that consists of two modules, short- and long-term temporal context (Fig. 1). The first module analyzes short-term spatio-temporal information and generates a probability value for every phase in each second of video by analyzing local information, both in the spatial and temporal domain. The second module is a long-term model that analyzes the entire video sequence by sequentially processing the first module predictions and outputs a single phase prediction for each second in the input.

The short-term model is based on inflating a Deep Convolutional Neural Network (DCNN) designed for 2D images into a 3D ConvNet model (I3D) designed for video action recognition^22^. In practice, this is done by adding an additional temporal dimension to the original network. In addition, in order to capture long-range temporal dependencies, we use non-local blocks within the I3D architecture^23^. The I3D model is obtained by converting a 2D image classification model. In this work, we follow Wang et al.^23^ and inflate a ResNet-50 model^27^. We use a pre-trained ResNet-50 weights, trained on ImageNet^24^ to initialize our I3D model weights, such initialization was shown to be beneficial when converting 2D models to their 3D counterpart^22^. Next, we train the model for the video action recognition task on the Kinetics-400 dataset^19^. This pre-training step is done in order to achieve a temporal baseline model on a large-scale video dataset. Finally, we finetune the baseline model by training on the surgery video dataset, replacing the last layer which is a 400-way classification layer with a randomly initialized 7-way layer to match the number of phases in laparoscopic cholecystectomy surgery^22,28^.

The short-term model is trained to predict a single phase for each second of the surgical video. We consider each second as an independent sample and the model input is a short 2.56 s clip around that second. As videos are encoded at 25 FPS this yields a clip of 64 images. We use a mini-batch size of 16 clips and train on a 4-GPU machine. Each mini-batch samples are randomly formed during training by randomly selecting a video and one of its seconds from the training set.

Each sample clip is first resized with its shorter side to 256 pixels. The input spatial size is randomly cropped during training and center cropped during evaluation to a 224 × 224 crop. Following a per channel mean and standard deviation normalization using preset ImageNet values (mean = [0.485, 0.456, 0.406] and std = [0.229, 0.224, 0.225]). Further augmentation is done during training, as part of producing the sample’s clip, by randomly selecting one anchor (middle) frame from the 25 possible frames within a second. Optimization is done using cross-entropy loss and Stochastic Gradient Descent (SGD) with an initial learning rate of 0.01 and a momentum of 0.9. The validation set is evaluated every 250,000 training samples and the learning rate is reduced three times by a factor of 10 after 10, 20 and 25 validation iterations and stop after 30 iterations. This translates into 2.5 million, 5 million, 6.25 million and 7.5 million training samples.

The model prediction errors can be divided into near errors, e.g. phase 3 is mistakenly predicted as phase 2, and distant errors, e.g. phase 0 is mistakenly predicted as phase 6. In order to overcome the latter one, we use a progress ratio and calculate a Second Duration Ratio (SDR) by dividing the sample second value with the overall video length. The SDR value is concatenated to the final layer before the classification layer. We found this addition to significantly reduce distant errors.

The final output of the short-term model is obtained from a SoftMax layer, which produces a probability vector per second, assigning a probability value for each phase. These vectors are then sequentially stacked together to create an L*7 matrix representation for each video, where L is the video length.

The long-term model is based on a Long Short-Term Memory (LSTM) network. This type of Recurrent Neural Network (RNN) uses special hidden units to maintain and accumulate memory from previous samples, enabling the model to learn long-term dependencies between samples^26,29^.

Here, a single input sample is considered as a sequence of phases values from an entire video. The fact that the input samples are an entire video sequence limits the training process to a relatively small number of samples. In order to avoid overfitting and apply data augmentation to such input, we use a SoftMax with temperature technique on the phases probability vectors^30^. We use a relatively high temperature value, T = 11, which produces a softer probability distribution. The probability vector is used to create a categorical distribution from which we randomly select a phase per second. The combination of categorical distribution phase selection and SoftMax with temperature considerably contributes to the model’s robustness and generalization.

The phases sequence is first processed with an embedding layer that works as a lookup table mapping each phase in the input to an embedding vector of size 32. Next, a single layer LSTM network with 128 hidden state features takes the embeddings as input. We use a bidirectional LSTM which uses past and future information to produce a final representation for each second in the input sequence. Finally, a linear layer operates as the classification layer and maps the output from the LSTM hidden space, back to the phases space. Cross-entropy loss is calculated for each second in the input video and model optimization is done with SGD using a fixed learning rate of 0.1 and a momentum of 0.9. We use a mini-batch of one since each video has a different sequence length and train the model for 30 epochs on a 1-GPU machine (an epoch is a single pass on the entire training samples).

Both models’ hyperparameters are first explored for the largest training dataset and remain fixed for all other experiments. For the short-term model, in case the training set number of available seconds is less than 250,000 the evaluation step is done every epoch and learning rate reduction is done in the same manner as before at 10, 20 and 25 validation iteration and stop at 30 iterations.

In the finetune for unseen medical center experiments we use only 15 training epochs in order to train the baseline model, reducing the learning rate after 5, 10 and 13 validation iterations. Although the number of training samples was 263,494 we ran an evaluation step every epoch. Fine-tuning on a different amount of training videos from medical center 1 is done in the same way, only initializing the models’ weights using the baseline model.

Our system is implemented with Python 3.6 using PyTorch (1.1.0) as the main deep learning framework^31^.

### Datasets

The short-term temporal model is first trained on the Kinetics-400 dataset^19^, this publicly available dataset contains about 240,000 training videos and about 20,000 validation videos. It was developed to facilitate research for human action classification and perform as a benchmark for building video classification models. Each video clip in Kinetics was curated from a different YouTube video and lasts around 10 s. It is labeled by a specific task, such as playing the clarinet, riding a bike, shaking hands, etc.

The backbone for the 3D temporal model^22^ is first trained as a 2D model^27^ for static image classification. This is done on the publicly available ImageNet dataset^24^. ImageNet contains about 1.3 million training images and 50,000 validation images from 1000 different classes.

In order to train the short- and long-term models for the phase detection task, we use a large laparoscopic cholecystectomy dataset. Our complete labeled data set consists of 1243 surgery videos curated from several sources, six different medical centers and more than 50 surgeons. It includes 80 videos from Cholec80^11^ and the rest from our internally proprietary dataset. Supplementary Tables S2 and S3 show detailed information about how surgeons and medical centers are distributed in the dataset.

Each video undergoes a rigorous annotation process by two different annotation specialists. Annotators are a group of medical students and surgeons who underwent thorough training on labeling the phases. A video is randomly assigned to a first annotator that reviews the video and manually assigns a surgical phase for each second of the video. The second annotator then validates the labels to reduce manual errors and enforce an aligned annotation process. In the case of unclear workflow or non-typical events, we also consult a group of surgeons in order to maintain high-quality labels. Annotation validity was confirmed by Korndorffer et al.^32^, in which an unbiased group of surgeons reviewed large portions of the cases in our dataset and reported high agreement with our annotations.

Although laparoscopic cholecystectomy is a relatively linear structured surgery, not all phases are mandatory to take place. We do force full covering of each video, i.e. each second is labeled with one and only one phase.

We apply several criteria for rejecting a video. We excluded videos in which surgery is done in a retrograde approach^33^ which is not a typical approach in our dataset, or those surgeries which eventually converted to open surgery. We tried to keep videos of both high and low quality, only excluding those in which the video is corrupted.

During the training and evaluation process, the video dataset is analyzed on a single second basis. Thus, the 1243 videos translate into more than 2.3 million second samples. Supplementary Table S1 shows statistical measurements of videos durations in the dataset.

The task of achieving an automated system that is able to recognize surgical phases with high accuracy has been explored in recent studies. However, all are limited to a small number of samples and lack the ability to generalize well to new samples^11–15^. The limitations mentioned in these previous studies are addressed in our work. We use a common practice in ML of dividing our dataset into a training set, composed of hundreds of videos, a different group of videos as a validation set to tune our models’ hyperparameters and another separate subset as an independent test set to further verify the results^26^. First, we randomly allocate 25% of the videos to serve as the independent test set. This test set is never seen during the training and hyperparameter tuning process and provides an unbiased evaluation set for the final model. The remaining videos are divided again into train and validation sets by an 80/20% ratio. Both short- and long-term models learn from the same train set and their hyperparameters are tunes on the same validation set. This setup yields a train, validation and test sets of 745, 187 and 311 videos, respectively. All subsets are randomly created on the video file level, thus each video takes part in only one subset. Both the short- and long-term models are trained on the same training set and hyperparameters are tuned on the same validation set.

## Results

**Figure 2:**
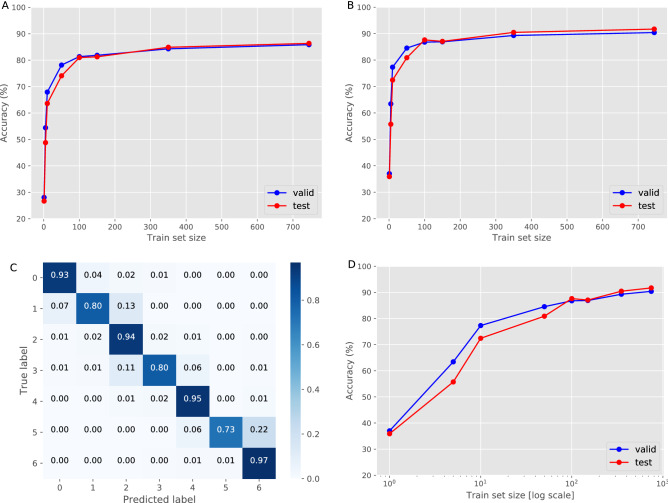
Asymptotic performance assessment. Accuracy values, measured on the validation and test sets, when training the models on an increasing number of videos (1, 5, 10, 50, 100, 150, 350, 745). (**A**) Accuracy for the short-term model. The log-scale version is available in Supplementary Fig. S1. (**B**) Accuracy for the long-term model. (**C**) The test set confusion matrix for the best long-term model. (**D**) Accuracy for the long-term model for a log-scale training set size.

**Figure 3:**
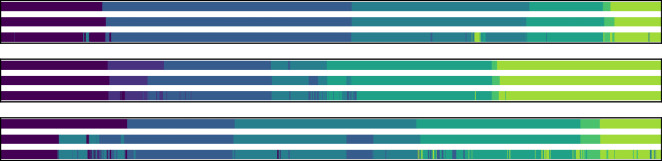
Comparing the true labels vs. model predictions. Three different videos from the test set are depicted. These videos represent the 90th percentile (top), median (middle) and 10th percentile (bottom) of the test set accuracy values. The top row of each video represents the sequence of true labels, the middle row represents the long-term model predictions and the bottom row represents the short-term model predictions. Each phase segment is illustrated with a different color.

**Figure 4:**
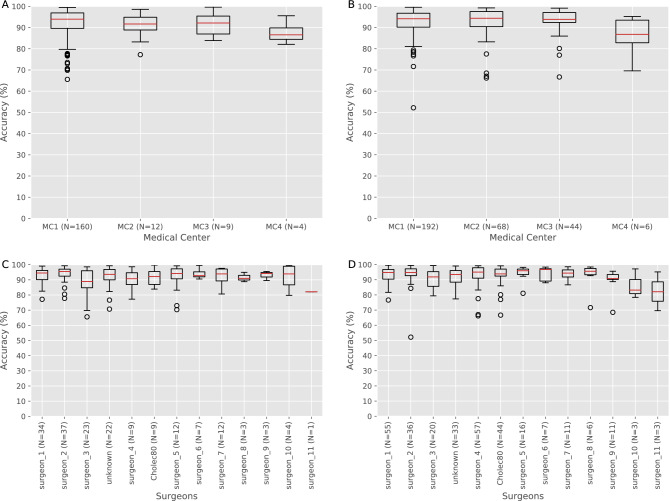
Medical centers and surgeons generalization evaluation. Accuracy boxplot for validation and test sets for different medical centers and surgeons. (N =) state the number of validation or test videos for each subset. (**A**) Validation set accuracy for each medical center (MC). (**B**) Test set accuracy for each medical center. MC3 is the Cholec80 dataset. Absolute number of procedures for each medical center is presented in Supplementary Table S2. Only MC1-MC4 are shown since MC5-6 have only one sample each. (**C**) Validation set accuracy for surgeons. (**D**) Test set accuracy for surgeons. Absolute number of procedures for each surgeon is presented in Supplementary Table S3. Only surgeons with more than 10 videos are analyzed. The red line marks the median value, the box extends from the lower to the upper quartile values, the whiskers extend from the box to show the range of the data (not including outlier) and the circle points are marking data outliers (outliers are based on 1.5IQR).

**Figure 5:**
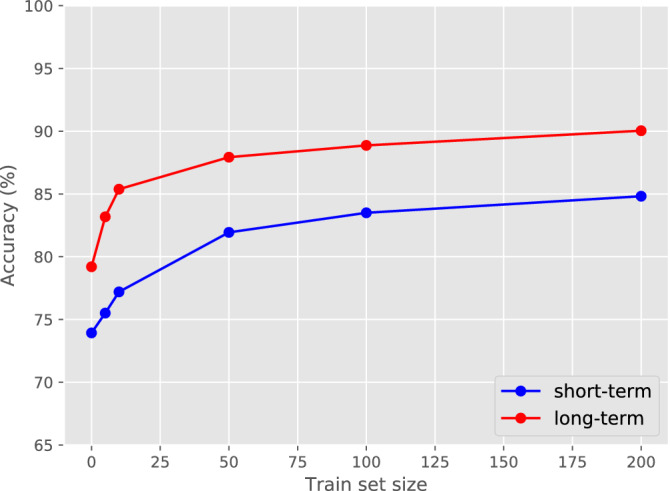
Finetune on a new medical center. Fine-tuning both the short- and long-term baseline models, which were initially trained on a dataset excluding videos from medical center 1, on an increasing number of videos from medical center 1. Log-scale version is available in Supplementary Fig. S2.

The main performance metric throughout this work is per-second accuracy. Each second of video footage is annotated with a single surgical phase. Accuracy is calculated by comparing the ground truth annotations with the system phase predictions (Fig. 1A–5). Accuracy is the ratio between the correct prediction and the overall number of seconds. Since phases do not distribute evenly during surgery (Supplementary Table S4) we also measure a mean phase accuracy, in which the accuracy of each phase is calculated separately (Fig. 2C) and a mean value over all phases is reported.

### Short-long temporal context modeling of surgical workflow

Our system has 90.4% accuracy and 86.1% mean phase accuracy on the validation set. On the test set it achieves 91.7% accuracy and 87.5% mean phase accuracy.

Classification errors can be generally grouped into three types: (1) A temporal shift where a predicted phase starts or ends early or late compared to the true labeled phase, (2) Adding or dropping entire phase segments, and (3) Random flips of a phase label. In order to better understand the nature of our system errors, we examine the confusion matrix (Fig. 2C) which illustrates each phase accuracy (diagonal) and its corresponding false rates (rows). Unsurprisingly, the model predominantly confuses temporally adjacent phases that share similar surgical context.

To more precisely assess the accuracy of phase transition, we establish a temporal window and test whether the predicted phase transition occurs with that window of the true transition. We perform this test for temporal windows (in seconds) between the prediction and the true start time of each phase for different τ values (τ =  ± 15, ± 30, ± 45, ± 60, ± 90, ± 120, ± 150, ± 180, ± 270, ± 360). Table 1 shows the percentage of phases for different τ values. Examining all videos in the validation and test sets shows more than 90% start time alignment at a temporal threshold of 45 s. However, this kind of analysis is affected by outlier procedures. Reviewing the failure modes of our system shows that some of the lowest accuracy videos are in fact unusual videos, e.g. a procedure which was converted to open surgery, or a video that was recorded using a single-port laparoscopic setup. Such outlier procedures are easy for a human to understand, however, for an ML system that was never trained on this type of samples it is not feasible. To reduce the effect of such cases on the phase transition accuracy we also examine a subset of videos with accuracy above the median accuracy (valid and test set per-video median accuracy is 93.67% and 94.15% respectively). This shows that, at a temporal threshold of 45 s, the alignment is above 98%. Examining a subset of videos with accuracy above the 10-percentile accuracy (valid and test set per-video 10-percentile accuracy is 83.15% and 82.93% respectively) shows an alignment of above 93% at 45 s. To complete the quantitative measurement we also show a qualitative comparison between the true labels and the predictions of our system (Fig. 3). This comparison shows one video from the 90th, median and 10th percentile of the test set results. The final prediction errors are mainly small temporal shifts in the phase start time. In the low accuracy video, our system also identifies false phase segments.

**Table 1:** Phase transition accuracy.

Temporal threshold	± 15	± 30	± 45	± 60	± 90	± 120	± 150	± 180	± 270	± 360
Valid	80.96	87.84	92.03	94.02	96.36	97.53	98.28	98.69	99.11	99.38
Test	79.95	87.63	91.81	93.73	95.99	97.23	98.04	98.51	99.23	99.4
Valid > 10-percentile	83.49	89.77	93.95	95.5	97.83	98.76	99.38	99.61	99.69	99.77
Test > 10-percentile	81.81	89.16	93.27	94.94	97.09	97.9	98.57	99.0	99.38	99.47
Valid > median	89.84	95.96	98.31	98.96	99.74	100	100	100	100	100
Test > median	88.83	95.48	98.34	98.8	99.45	99.54	99.63	99.72	99.82	99.91

Evaluating the effect of small temporal shifts in phase start time. Testing whether a predicted phase transition occurs within a temporal threshold (in seconds) of the true transition. Numbers state the percentage of phases within the threshold. The first two rows are showing all valid and test videos, the last two rows are showing only a subset of videos with per-video accuracy greater than the videos median accuracy and the middle two rows are done on a subset without including the bottom 10% videos.

The best performing hyperparameters established on the validation set were used for all model training and testing throughout this work. Initially this was done in order to enable a fair comparison between models, but we found this set of hyperparameters an important result of our work, making it possible to use our approach and train models on relatively small publicly available datasets, without the need of a complex and long hyperparameters exploration on large train and validation sets.

### Asymptotic performance assessment

Our first question was how many surgical videos are needed to reliably recognize the phases of surgery. We approach this by examining the change in the system accuracy on the test set as a function of training data size. The validation and test sets are kept fixed in order to maintain a fair comparison. Models’ hyperparameters were first optimized for the largest training dataset and remain fixed for all other experiments.

We start by examining the short-term context model, training the model on a different number of videos, ranging from a single video up to 745 videos (Fig. 2A). The model reaches accuracy greater than 80% at 100 videos and keeps increasing as the number of samples grows, reaching a near asymptotic value of more than 85% at the endpoint of 745 training videos. Next, the predictions of the short-term context model are used to train the long-term model (Fig. 1). This model reaches 80% accuracy at only 50 training videos and achieves more than 90% accuracy at 745 training videos (Fig. 2B,D). The validation set results are slightly higher than those of the test set when training on less than 100 videos. This is expected because the validation set is used for model hyperparameter tuning and is thus biased. However, the test set is kept completely independent and represents a blind evaluation set. The gap decreases as the number of samples in the training set increases, implying the importance of training such a system on a large dataset and the impact it has on the robustness and generalization of the proposed method to new unseen samples.

Figure 2D presents the system’s accuracy as a function of the number of training videos on a log-scale. This clearly shows that there is a substantial increase in performance when training on hundreds of videos compared to only tens of videos as done in previous studies. Once reaching near a thousand training videos, we are able to cross the 90% accuracy. However, extrapolating from these results, it appears that increasing the number of training videos an order of magnitude (to 10′s of thousands) would yield small fraction (1–2%) of remaining performance improvement. If the same trend continues for an additional order of magnitude (to 100′s of thousands of videos), it would appear that nearly all of the practical value of training is achieved with more than 100 but fewer than 1000 videos, for the current model setup.

### Model generalization: medical center related bias

The question of how many videos are needed for surgical phase recognition can also be examined as a generalization question. Namely, how effectively does the system generalize to various data sources—i.e. surgeons or medical centers.

We first consider the effect of data imbalance related to medical centers (Supplementary Table S2). Our dataset is skewed toward medical center 1 (MC1). This might affect the results of other medical centers within the dataset. However, videos from medical center 1 were curated over decades and span various surgery styles, different surgeons and a variety of recording systems. In order to assess if such bias exists we evaluate the accuracy of the best model, trained on 745 videos, for each medical center in our validation and test sets (Fig. 4A,B). The results show relatively consistent performance for all medical centers in the validation and test sets. Medical center 4 has the lowest performance. This might be related to the fact that it had only 23 videos in the training set. In addition, the accuracy per medical center is similar to the overall accuracy of the model suggesting that the model generalized well to a variety of different medical centers.

### Model generalization: surgeon related bias

Here we explore whether our model is biased toward specific surgeons. The dataset includes 36 different surgeons (1,042 videos), Cholec80 group^11^ (80 videos) which includes 13 additional surgeons (without a surgeon labeling per video) and an additional group (121 videos) missing surgeon labeling (unknown group). In the analysis, we focus on 11 different surgeons, the Cholec80 group and the unknown group. These 13 subsets have more than 10 videos in the dataset (Supplementary Table S3).

We measure the accuracy of each surgeon separately within the validation and test sets (Fig. 4C,D). Most of the surgeons in our comparison show similar results in the validation and test set. The last three surgeons in the analysis had very few video samples in either the validation or test set so their statistical significance is questionable. The majority of the surgeons’ individual accuracy is consistent with the overall performance of the system, demonstrating the robustness of the model to a variety of surgeons and different operating techniques.

### Model adaptation: fine-tuning on a new medical center

Model adaptation is an important capability that enables fast deployment in new surgical departments and medical centers. Adapting models to a new unseen medical center can be done using a finetuning approach, in which the learning process continues on a relatively small number of new samples^28^. In the scope of this work, as the dataset is skewed toward MC1, which has 1002 videos, it is interesting to examine the effect of discarding MC1 samples during training. We explore how the largest medical center affects the overall performance and whether there is a bias toward that specific medical center.

The original dataset is divided into two subsets. The first is formed by discarding all MC1 videos which yields a training set of 95 videos and a test set of 119 videos. The second subset contains only videos from MC1 and has 650 training videos and 192 test videos.

Next, new baseline models were trained on the first subset. The short- and long-term models achieve 83.15% and 89.6% accuracy on the first subset test set, respectively. However, these models produce only 73.9% and 79.2% accuracy on the second subset (which has only MC1 videos). This shows degradation of about 10% compared to the first subset, and is consistent with what we expect to see on smaller than 100 training videos (Fig. 2A,B).

We adapt the baseline models to the MC1 by continuing the learning process of both models. In order to evaluate how many samples are needed, we use an increasing number of training samples from MC1, ranging from 5 to 200, fine-tuning the baseline models and measure the performance on the second subset test set (Fig. 5).

The short-term model achieves more than 80% accuracy after only 50 new videos and was able to reproduce the original result at the 200 videos (Fig. 5, Supplementary Fig. S2). The long-term model shows the same trend, able to achieve an 88% accuracy after the same 50 new videos and 90% accuracy at 200 new videos (Fig. 5, Supplementary Fig. S2).

This experiment further supports the fact that training with only tens of videos is not sufficient to achieve a generalized model and handle unseen samples. The fine-tuned model reaches 90% accuracy only after training on hundreds of new videos from MC1.

## Discussion and conclusions

In this work, we follow previously suggested guidelines for ML to overcome “barriers to deployment” and have an actual impact on healthcare^34^. Our study aims to support the transition from bench to bedside and help translate research into tools that aid surgeons, practically, in their daily routine. The viewpoint by Wang et al.^35^ discusses the challenges of integrating deep learning applications into the clinician’s workflow. Challenges such as data quantity and quality and model generalizability are addressed in this study. Since our dataset is more than an order of magnitude larger than any previous study it provides in-depth knowledge of surgical workflow characteristics. In addition, as ML systems are often considered as a “black-box”, we train our approach based on predefined phase mapping, agreed to by a group of experts in the field. This ensures that healthcare professionals understand and can interpret the results.

In summary, the contributions of this work are threefold. First, we leverage a large and diverse surgical video dataset and apply state-of-the-art methods for video analysis to introduce a deep learning system that achieves more than 90% accuracy in detecting the correct surgical phase. In order to support future research in this field, we report the set of hyperparameters learned and optimized with our dataset, allowing to train models on smaller but publicly available datasets. Second, we report a comprehensive analysis, assessing the likely asymptotic performance of our phase detection system and evaluating the generalization of our approach to different medical centers and a diverse surgeon population. Third, given a set of unseen videos from a new medical center, we demonstrate that by fine-tuning on a relatively small number of new samples, our model can converge to a similar high performance.

Examining the number of videos needed in order to learn the surgical workflow shows high return in performance when going from tens to hundreds of training videos, where a diminishing effect in accuracy is starting to appear. We do note that our performance assessment was done using a fixed model capacity. Increasing the available data to thousands or even tens of thousands of videos may offer additional improvements by also increasing the model capacity.

As opposed to elite athletes who can review performance and analyze every roll, pitch and yaw post-competition, surgeons lack objective tools to routinely debrief and analyze performance. The current level of accuracy allows for the creation of surgical highlight reels that can be returned to a surgeon quickly after exiting the operating room. Higher levels of accuracy will be required in order to leverage such capabilities for real-time decision support.

The results in this report are limited to one specific surgical procedure, laparoscopic cholecystectomy, which has a relatively linear phase progression. Further analysis is needed in order to ensure the transferability of the ideas presented here to less structured or lengthy procedures. In addition, both modules do not assume any causality constraints and design to operate offline on the entire surgical video. However, enforcing such constraints can easily be done by adjusting the models to “look” at past inputs only. The evaluation of such models is not within the scope of this work. Although procedures from MC1 were curated over decades and capture high surgical variability, the dataset is still skewed towards this single center. Our generalization exploration focuses on high-level biases, which might occur due to different medical centers, surgeons and techniques. Once sufficiently labeled, future exploration should be done to assess both patient-level bias factors such as age, BMI, ethnicity, sex, anatomy variance, etc. and medical-center-level bias, e.g. by grouping procedures based on visualization hardware, instruments, or on the period or date of performing the surgery. Our models are also limited in handling outliers, such as very low video quality or in case a new surgical tool, never seen before, is used.

The hyperparameters described for the short- and long-term approach enable training robust models on relatively small, but publicly available, datasets such as Cholec80^11^ and EndoTube^36^. The ability to detect surgical phases with high accuracy can promote the development of other surgical applications and support continuous research in this field. In addition, robust phase detection models can be the catalyst for studies of intraoperative event detection or applying transfer learning on other types of laparoscopic procedures.

There are many opportunities for ML-based AI in the operating room^10^. Regardless of the use case, a foundational step in integrating AI systems into routine surgeon workflow is the ability to analyze and successfully discern between different surgical phases. Thus, surgical phase detection is a key benchmark problem to assess the ability of AI to successfully learn surgical context.

We believe our analysis can further accelerate computer vision-based research and applications for laparoscopic surgery to the point of integrating AI systems in the surgical workflow routine, assisting in the decision-making processes and ultimately improving surgeon experience and patient care.

## Supplementary Information

Supplementary Information.

## Data Availability

The data that support the findings of this study constitute of published data and restricted data. Published data are available from the reported references. Restricted data are under a non-published license and are not publicly available.
