# Supplementary material for: Impact of data on generalization of AI for surgical intelligence applications
Source: Sci Rep. 2020 Dec 17;10:22208. doi: 10.1038/s41598-020-79173-6 (PMC7747564; doi:10.1038/s41598-020-79173-6)
Supplement: Supplementary file 1 — Supplementary Information. [file 41598_2020_79173_MOESM1_ESM.pdf]

Supplementary Materials

Impact of Data on Generalization of AI for Surgical Intelligence Applications

Omri Bar[1]*, Daniel Neimark[1], Maya Zohar[1], Gregory D. Hager[1,2], Ross Girshick[1],
Gerald M. Fried[1,3], Tamir Wolf[1], Dotan Asselmann[1]

[1]theator Inc., San Mateo, CA, USA.

[2]Department of Computer Science, Johns Hopkins University, Baltimore, USA.

[3]Department of Surgery, McGill University Health Centre, Montreal, QC, Canada.

*e-mail: omri@theator.io

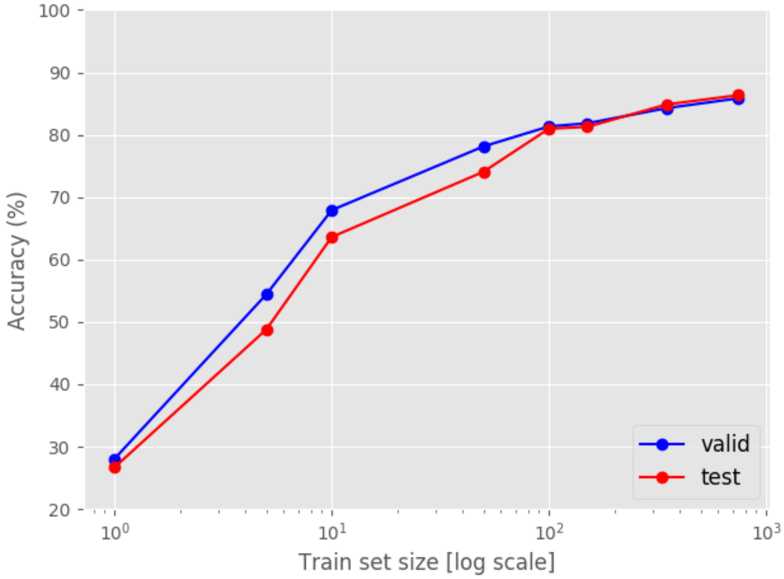

Supplementary Figure S1: Short-term model accuracy values for different number of training videos, presented in log-scale.

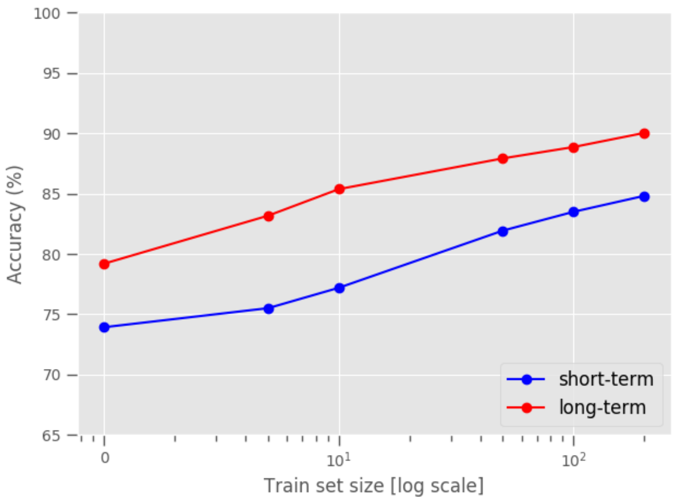

Supplementary Figure S2: Fine-tuning the baseline models on an increasing number of videos from medical center 1, presented in log-scale.

	Number of Videos	Duration Mean	Duration STD	Duration Min	Duration Max	Duration Median
Full dataset	1243	0:32:14	0:19:03	0:05:11	3:17:21	0:27:33
Train	745	0:32:14	0:19:54	0:05:11	3:17:21	0:27:12
Valid	187	0:31:17	0:17:44	0:07:54	1:44:33	0:26:50
Test	311	0:32:49	0:17:41	0:05:49	2:46:45	0:28:29

Supplementary Table S1: Statistical measurement of videos duration in the full dataset and in each one of the subsets.

	Full dataset	Train	Valid	Test
Medical Center 1	1002	650	160	192
Medical Center 2	123	43	12	68
Medical Center 3	80	27	9	44
Medical Center 4	33	23	4	6
Medical Center 5	3	1	1	1
Medical Center 6	2	1	1	0

Supplementary Table S2: Number of videos for each medical center in the full dataset and each one of the different subsets. Medical Center 3 is the Cholec80 dataset.

	Full dataset	Train	Valid	Test
surgeon_1	289	200	34	55
surgeon_2	218	145	37	36
surgeon_3	124	81	23	20
Unknown	121	66	22	33
surgeon_4	104	38	9	57
Cholec80 (13 surgeons)	80	27	9	44
surgeon_5	71	43	12	16
surgeon_6	46	32	7	7
surgeon_7	46	23	12	11
surgeon_8	45	36	3	6
surgeon_9	19	5	3	11
surgeon_10	16	9	4	3
surgeon_11	10	6	1	3
surgeon_12	9	7	2	0
surgeon_13	3	3	0	0
surgeon_14	3	1	1	1
surgeon_15	3	3	0	0
surgeon_16	3	1	2	0
surgeon_17	3	2	0	1
surgeon_18	3	1	1	1
surgeon_19	3	1	2	0
surgeon_20	2	2	0	0
surgeon_21	2	2	0	0
surgeon_22	2	1	0	1
surgeon_23	2	2	0	0
surgeon_24	2	1	1	0
surgeon_25	2	1	0	1
surgeon_26	2	0	0	2
surgeon_27	1	1	0	0

surgeon_28	1	1	0	0
surgeon_29	1	1	0	0
surgeon_30	1	1	0	0
surgeon_31	1	1	0	0
surgeon_32	1	1	0	0
surgeon_33	1	0	1	0
surgeon_34	1	0	1	0
surgeon_35	1	0	0	1
surgeon_36	1	0	0	1

Supplementary Table 3: Number of videos for each surgeon for the full dataset and each one of the different subsets. Surgeon's names are anonymized for privacy reasons. One record is named "Unknown" representing videos in our dataset for which the specific surgeon was not mentioned in the metadata. In addition, one of the records represents Cholec80 dataset which is known to have 13 surgeons. Our dataset includes 1,042 videos from 36 known surgeons, 80 videos from 13 Cholec80 surgeons and 121 videos from a group of unknown surgeons.

Phase	Full dataset	Train	Valid	Test
Phase 0 - Preparation	1181	708	178	295
Phase 1 - Adhesiolysis	792	485	123	184
Phase 2 - Dissection	1242	744	187	311
Phase 3 - Division	1242	744	187	311
Phase 4 - Separation	1242	744	187	311
Phase 5 - Packaging	1021	604	148	269
Phase 6 - Final inspection	1231	737	187	307

Supplementary Table S4: Phase distribution in the dataset and per subset. Phases are counted in each video with repetition, in case a phase appears more than once in a procedure all of his instances will be counted.
